# Molecular Characterisation of Aflatoxigenic and Non-Aflatoxigenic Strains of *Aspergillus* Section *Flavi* Isolated from Imported Peanuts along the Supply Chain in Malaysia

**DOI:** 10.3390/toxins11090501

**Published:** 2019-08-29

**Authors:** Mahror Norlia, Selamat Jinap, Mahmud Ab Rashid Nor-Khaizura, Son Radu, Cheow Keat Chin, Nik Iskandar Putra Samsudin, Abdul Halim Farawahida

**Affiliations:** 1 Department of Food Science and Technology, Faculty of Food Science and Technology, Universiti Putra Malaysia, UPM Serdang, Selangor 43400, Malaysia; 2 School of Industrial Technology, Universiti Sains Malaysia, Minden 11800, Pulau Pinang, Malaysia; 3 Laboratory of Food Safety and Food Integrity, Institute of Tropical Agriculture and Food Security, Universiti Putra Malaysia, UPM Serdang, Selangor 43400, Malaysia; 4 Food Safety and Quality Division, Ministry of Health Malaysia, Putrajaya 62675, Malaysia

**Keywords:** peanuts, aflatoxins, *Aspergillus flavus*, aflatoxin biosynthesis gene

## Abstract

Peanuts are widely consumed in many local dishes in southeast Asian countries, especially in Malaysia which is one of the major peanut-importing countries in this region. Therefore, *Aspergillus* spp. and aflatoxin contamination in peanuts during storage are becoming major concerns due to the tropical weather in this region that favours the growth of aflatoxigenic fungi. The present study thus aimed to molecularly identify and characterise the *Aspergillus* section *Flavi* isolated from imported peanuts in Malaysia. The internal transcribed spacer (ITS) and β-tubulin sequences were used to confirm the species and determine the phylogenetic relationship among the isolates, while aflatoxin biosynthesis genes (*aflR*, *aflP (omtA)*, *aflD (nor-1)*, *aflM (ver-1)*, and *pksA*) were targeted in a multiplex PCR to determine the toxigenic potential. A total of 76 and one isolates were confirmed as *A. flavus* and *A. tamarii*, respectively. The Maximum Likelihood (ML) phylogenetic tree resolved the species into two different clades in which all *A. flavus* (both aflatoxigenic and non-aflatoxigenic) were grouped in the same clade and *A. tamarii* was grouped in a different clade. The aflatoxin biosynthesis genes were detected in all aflatoxigenic *A. flavus* while the non-aflatoxigenic *A. flavus* failed to amplify at least one of the genes. The results indicated that both aflatoxigenic and non-aflatoxigenic *A. flavus* could survive in imported peanuts and, thus, appropriate storage conditions preferably with low temperature should be considered to avoid the re-emergence of aflatoxigenic *A. flavus* and the subsequent aflatoxin production in peanuts during storage.

## 1. Introduction

*Aspergillus* section *Flavi* is one of the most important sections in the genus *Aspergillus* as the majority of the species in this section are able to produce aflatoxins, of which aflatoxin B_1_ (AFB_1_) is a carcinogenic compound that can cause acute and chronic diseases related to aflatoxin poisoning [1]. Acute exposure of aflatoxin may lead to death as reported in Kenya in 2004 [2], while chronic exposure may lead to liver cancer [3]. AFB_1_ has been classified as a Group 1 carcinogen by the International Agency of Cancer Research [4]. According to [5], *Aspergillus* section *Flavi* could be separated into two groups based on their impact on food and human health. The first group includes the main aflatoxigenic species such as *A. flavus*, *A. parasiticus* and *A. nomius*, while the second group comprises the non-aflatoxin-producing species such as *A. tamarii*, *A. oryzae* and *A. sojae.* These are the main important species found in crops—especially in nuts, spices, cereals and also fermented product such as *meju* [6,7,8,9,10]. These species are closely related to each other in terms of morphology and phylogeny [5,11]. However, *A. flavus* is reported to be more diverse in terms of morphological characters and toxigenic potential [12,13].

Molecular and phylogenetic analyses are commonly used to validate the morphological identification of *Aspergillus* section *Flavi*. The DNA sequence of the conserved regions in fungi, especially in the genus *Aspergillus* such as the ITS regions, β-tubulin and calmodulin, could be used to differentiate the closely related species of *Aspergillus* section *Flavi* such as *A. parasiticus*, *A. oryzae*, *A. minisclerotigenes*, *A. parvisclerotigenus* and *A. arachidicola* [14,15]. 

The occurrence of aflatoxin and *Aspergillus* spp. in raw peanuts (imported) and peanut-based products marketed in Malaysia have been documented since 1980s [16] and the contamination has been increasing along the supply chain, especially at the level of manufacturers and retailers as reported by [17]. The authors [17] then reported on the presence of aflatoxigenic and non-aflatoxigenic *A. flavus* and one isolate of *A. nomius* from the same peanut samples based on the morphological and chemical (extrolites) characteristics [18]. Based on the findings, some of the aflatoxigenic *A. flavus* were able to produce both aflatoxin B and G group aflatoxins which are the common features in other species in section *Flavi* such as *A. parasiticus*, *A. arachidicola* and *A. minisclerotigenes* [19]. In a previous study, *A. tamarii* and *A. nomius* were misidentified as *A. flavus* due to the phenotypic resemblance as reported by [20]. In this regard, the misidentification of aflatoxigenic and non-aflatoxigenic *A. flavus* could have occurred due to these similarities and thus requires additional molecular data to support the results [18], which is presented and discuss in this paper.

Even though the *Aspergillus flavus* and *A. parasiticus* agar (AFPA) medium was specifically formulated by [21] to isolate and enumerate *A. flavus* and *A. parasiticus* from food samples, recent studies have reported that other *Aspergillus* section *Flavi* species were also capable of producing aspergillic acid, which could react with the ferric citrate in the medium to produce orange colour reverse [14,22,23]. Therefore, gene sequencing and phylogenetic analysis were required to resolve and confirm the identification of *Aspergillus* section *Flavi* species. The ITS region has been widely used as the ‘barcode’ for fungal identification [24]. However, depending on a single gene identification is not always accurate due to the intra- and inter-species variation of the *Aspergillus* spp. The analysis of DNA sequences from two or more genes are thus deemed more accurate and reliable. Therefore, β-tubulin was used as the secondary identification marker in the present study to validate the species identification [25].

The aflatoxin-producing ability of *A. flavus* in the previous study [18] was found to be inconsistent since a large number of strains were found to be non-aflatoxigenic. Other than the environmental conditions, the ability of *A. flavus* to produce aflatoxin is highly determined by the genetic variation of the strains. It could be due to any disruption in the aflatoxin biosynthesis genes or belong to other species that do not produce aflatoxin. The aflatoxin biosynthesis gene cluster has been sequenced and extensively studied in order to understand the mechanism and biosynthesis pathway of aflatoxin in aflatoxigenic fungi [26]. The presence of these genes is required by the *Aspergillus* spp. to produce aflatoxin, and any changes therein might cause disruption in the biosynthetic pathway. The precise identification of *Aspergillus* section *Flavi* is therefore important to determine the risk of aflatoxin contamination as results in the previous study [17,18] indicated a high occurrence of these species in raw peanuts (imported) and peanut-based products marketed in Malaysia. 

Therefore, the objectives of the present study were to molecularly confirm the identity of *A. flavus* and *A. nomius* from the previous study [18], to determine the phylogenetic relationships among the *Aspergillus* section *Flavi* strains, and to detect the presence of aflatoxin biosynthesis genes in those strains.

## 2. Results

### 2.1. PCR Amplification and Basic Local Alignment Search Tool (BLAST) Search

The PCR amplifications of the ITS region and β-tubulin genes for all strains were positive, generating products of ~600 bp and ~595 bp, respectively. Based on the BLAST search against the GenBank database, both ITS and β-tubulin genes gave a similar result for all 77 *Aspergillus* section *Flavi* strains in this study (Table 1). Results from the ITS and β-tubulin gene sequencing are in line with the previous identification of *A. flavus* except for A52R. The BLAST results showed that a total of 76 strains were identified as *A. flavus*/*A. oryzae* with 99 to 100% similarity, while A52R, which was previously identified as *A. nomius*, was re-identified as *A. tamarii* based on the ITS and β-tubulin gene sequences. The DNA sequences analysis confirmed the absence of *A. parasiticus* in raw peanuts and peanut-based products tested in the previous study [18]. A total of 37 out of 46 (92.5%) aflatoxigenic *A. flavus* (Chemotype I, II, and V) were isolated from raw peanut kernels in which 57 and 35% of the them were imported from India and China, respectively (Table 1).

### 2.2. Phylogenetic Analysis

**Figure 1:**
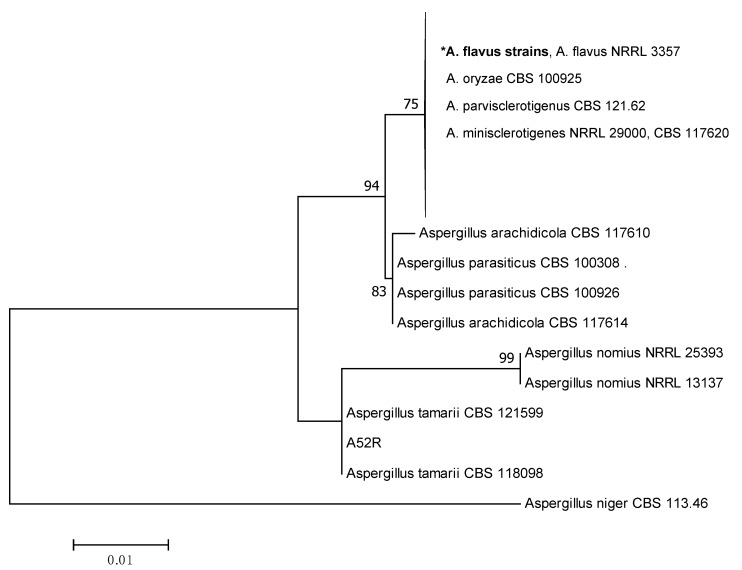
Maximum likelihood tree showing the phylogenetic relationships among the *Aspergillus* section *Flavi* strains based on the ITS sequences. *A. flavus includes all strains in Chemotypes I—V as listed in Table 1. Values on branches are the bootstrap values.

**Figure 2:**
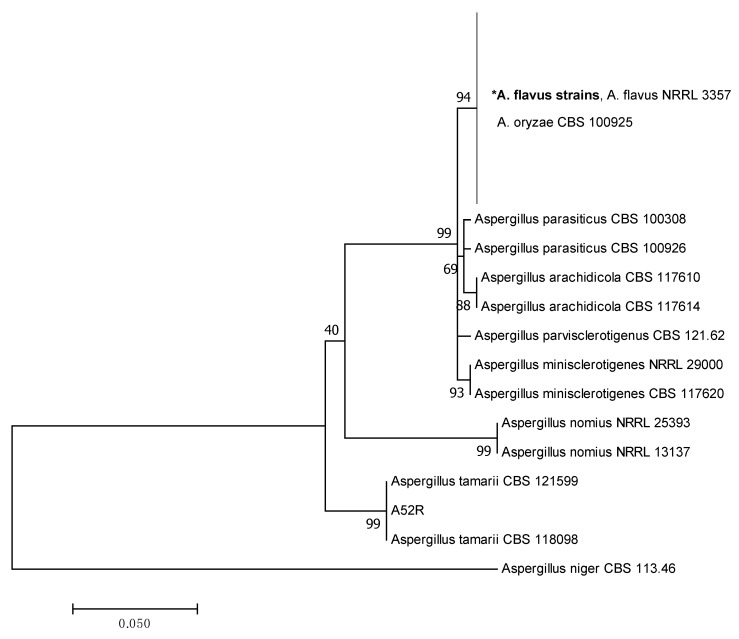
Maximum likelihood tree showing the phylogenetic relationships among the *Aspergillus* section *Flavi* strains based on the β-tubulin sequences. *A. flavus includes all strains in Chemotypes I—V as listed in Table 1. Values on branches are the bootstrap values.

**Figure 3:**
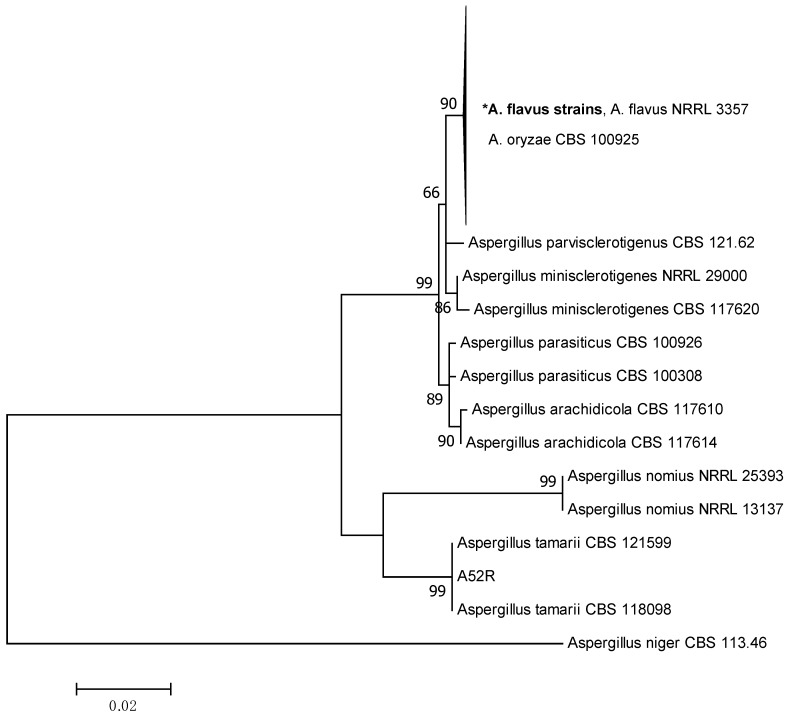
Maximum likelihood tree showing the phylogenetic relationships among *Aspergillus* section *Flavi* strains based on the combined ITS and β-tubulin sequences. *A. flavus includes all strains in Chemotypes I—V as listed in Table 1. Values on branches are the bootstrap values.

The Maximum Likelihood (ML) tree was constructed based on the ITS, β-tubulin and combined sequences to describe the phylogenetic relationships among the *Aspergillus* section *Flavi* strains as shown in Figure 1, Figure 2 and Figure 3, respectively. The individual ML tree for ITS sequences does not clearly separate different species into different clades as shown in Figure 1. The reference strains of *A. flavus*, *A. oryzae*, *A. parvisclerotigenus* and *A. minisclerotigenes* were grouped together in the same clade. This result demonstrated that ITS alone is not enough to resolve the closely related species of *Aspergillus* section *Flavi*. However, β-tubulin and the combined ITS and β-tubulin sequences showed better separation of each species into different clades and were supported with medium to high bootstrap values ranging from 60–99%.

The ML tree for β-tubulin, as shown in Figure 2, grouped 76 strains in the present study in the same clade with the reference strains *A. flavus* NRRL 3357 and *A. oryzae* CBS 100925. Both reference strains could not be separated due to the high genetic similarity in both species [27]. However, the identities of the strains in this group were confirmed as *A. flavus* as they originated from peanuts and the majority of them showed the ability to produce aflatoxins (Table 1). In contrast, *A. oryzae* does not produce aflatoxin, and it has never been reported in peanuts [28]. The species is mainly used in koji fermentation for traditional fermented food in Japan. On the other hand, one isolate of *A. tamarii* was consistently grouped together with the reference strains of *A. tamarii* CBS 121599 and CBS 118098. 

The ML tree of the combined dataset (Figure 3) shows similar tree topology with the individual β-tubulin. All strains were grouped together with the reference strains *A. flavus* NRRL 3357 and *A. oryzae* CBS 100925 except for A52R which was grouped together with *A. tamarii* CBS 121599 and *A. tamarii* CBS 113.46. The outgroup *A. niger* CBS 113.46 formed a separate clade. 

Generally, the *A. flavus* strains in the present study were clustered in the same clade and not according to the source of isolation. *A. flavus* strains isolated from raw peanuts and peanut-based products collected from different stakeholders did not show any genetic variation as they were consistently grouped in the same clade. Furthermore, the aflatoxigenic and non-aflatoxigenic *A. flavus* did not form a separate clade, since ITS and β-tubulin genes were mainly used for identification purposes, and they were not involved in the biosynthesis of aflatoxin.

### 2.3. Detection of Aflatoxin Biosynthesis Genes in Aspergillus Section Flavi Strains

**Figure 4:**
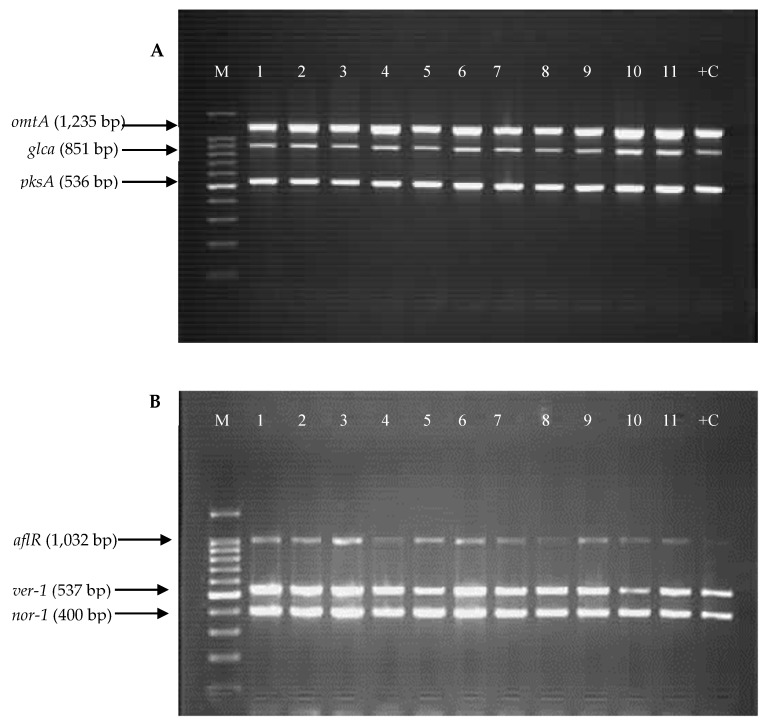
Amplification of (**A**) Multiplex PCR set 1: *omtA*, *glca*, and *pksA* and (**B**) Multiplex PCR set 2: *aflR*, *ver-1* and *nor-1* genes in the representative aflatoxigenic *A. flavus* (Chemotype V). M: 100-bp DNA ladder; Lane 1: A1R; Lane 2: A15R; Lane 3: A25R; Lane 4: A29R; Lane 5: A41R; Lane 6: A44R; Lane 7: A69R; Lane 8: A80R; Lane 9: A82R, Lane 10: A102R; Lane 11: A107R and Lane 12: +C Positive control (*A. flavus* NRRL 3357).

The PCR method was used to amplify the targeted aflatoxin biosynthesis genes *aflR, aflP (omtA), aflD (nor-1), aflM (ver-1), pksA*, and one sugar utilisation gene, *glcA.* The *glcA* gene, which is located adjacent to the 3′ end of aflatoxin biosynthesis gene cluster, was used as a positive marker for *A. flavus*, as this gene is consistently present in this species regardless of toxigenic potentials [29,30]. Figure 4A,B show the representative amplification patterns of the reference strain *A. flavus* NRRL 3357 and the aflatoxigenic *A. flavus* isolates from Chemotype V in Multiplex PCR set 1 and 2, respectively. All the targeted genes were successfully amplified and corresponded to the sizes of their PCR products. The results support the ability of these strains to produce aflatoxin. 

**Figure 5:**
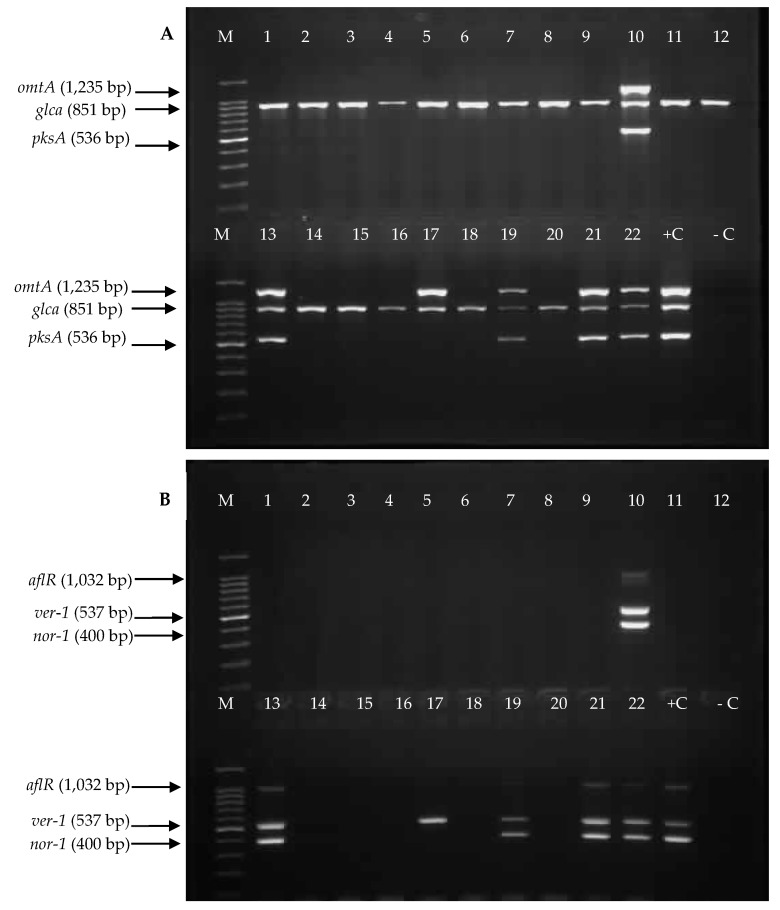
Representative amplification of (**A**) multiplex PCR set 1: *omtA*, *glca*, and *pksA* and (**B**) Multiplex PCR set 2: *aflR*, *ver-1* and *nor-1* genes in the representative non-aflatoxigenic *A. flavus* **(Chemotype IV).** M: 100-bp DNA ladder; +C: Positive control (*A. flavus* NRRL 3357); -C: Negative control (without DNA template); Lane 1: A9R; Lane 2: A12R; Lane 3: A13R; Lane 4: A14R; Lane 5: A16R; Lane 6: A19R; Lane 7: A20R; Lane 8: A21R; Lane 9: A22R; Lane 10: A23R; Lane 11: A26R; Lane 12: A27R; Lane 13: A67R; Lane 14; A75R; Lane 15: A76R; Lane 16: A98R; Lane 17: A104P; Lane 18: A111P; Lane 19: A114P; Lane 20: A115P; Lane 21: A122R; Lane 22: A123R.

In contrast, the representative non-aflatoxigenic *A. flavus* strains from Chemotype IV failed to amplify almost all the genes required for aflatoxin biosynthesis as shown by the results of Multiplex PCR set 1 and set 2 in Figure 5A,B, respectively. The amplification patterns of the aflatoxin biosynthesis genes in all *A. flavus* strains and *A. tamarii* in the present study are summarised in Table 2. At least one gene was missing as depicted by the amplification patterns that caused the strains to fail to produce aflatoxin except for A23R, A67R, A122R and A123R in Chemotype IV. The majority (59%) of the non-aflatoxigenic strains in Chemotype IV that failed to amplify all the genes originated from raw peanut samples collected from the importers. However, all non-aflatoxigenic *A. flavus* in Chemotype III showed a complete amplification of all genes except for A40R, A43R and A48R. As a comparison, the *glcA* gene was amplified in all strains, as this gene is not involved in the aflatoxin biosynthetic pathway. In addition, the complete amplification pattern in reference strain *A. flavus* NRRL 3357 confirmed that the absence of these genes in the non-aflatoxigenic *A. flavus* isolates was not caused by any technical error.

## 3. Discussion

A comparison of both ITS and β-tubulin sequences with fungal sequences deposited in the GenBank showed a high similar percentage for *A. flavus* and *A. oryzae.* The strong phylogenetic relationship between *A. flavus* and *A. oryzae* has been explained by many researchers and they concluded that *A. oryzae* is actually the domesticated species of *A. flavus* through years of selection under artificial production environments [27,31,32,33]. *A. oryzae* has been widely used for commercial application such as the starter culture for koji fermentation in the production of traditional fermented foods such as soy sauce, sake and shochu [34], and it has earned the Generally Regarded as Safe (GRAS) status due to its long history of safe use in the food fermentation industry. Payne et al. [27] who studied the whole genome comparison of *A. flavus* and *A. oryzae* revealed that these fungi are very similar in the genome size and number of predicted genes. However, due to the economics and food safety issues, *A. oryzae* continues to be classified as a separate species from *A. flavus* even though it has been proven to be genetically similar to *A. flavus* [27,31]. 

*A. oryzae* is not a plant pathogen and it has never been reported to contaminate peanuts in the field [35,36,37]. It is believed that *A. oryzae* rarely survives in the field due to the low production of sclerotia, which could be detrimental to its survival [31,33]. According to [19], no aflatoxin production has been recorded from this species. Besides, a study on the comparative chemistry of *A. flavus* and *A. oryzae* also revealed that the latter species does not produce CPA [38]. Therefore, *A. flavus* was confirmed as the main aflatoxigenic and non-aflatoxigenic strains detected in raw peanuts and peanut-based products in the present study. Interestingly, *A. parasiticus* was absent in the present study even though it was reported as one of the main aflatoxin producers in peanuts by previous researchers [6,35,37].

Peanuts were dried to achieve a moisture content of <9% during post-harvest, and this condition is maintained throughout the shipping period to the importing countries to prevent fungal proliferation. However, the ability of *A. flavus* to produce sclerotia, which is a compact mass of hardened mycelium that contains food reserves, helps them to survive in the extreme environmental conditions until favourable growth conditions return [39,40].

In the previous study [18], one strain (A52R) has been morphologically identified as *A. nomius* due to the production of AFB, AFG, aspergillic acid and limited growth on CZ agar at 42 °C. However, molecular analysis based on the sequences of ITS and β-tubulin region revealed the identity as *A. tamarii*. It was found to be an unusual observation of *A. tamarii* since it was able to produce aflatoxins, which is contradictory to its typical characteristics. According to [15], *A. tamarii* does not produce aflatoxins, and it has been used in the food industry for the production of soy sauce and various enzymes such as amylases, proteases and xylanolytic enzymes since a long time ago.

However, an isolated case was reported for the first time by [41], in which several strains of *A. tamarii* isolated from a tea field were found to produce aflatoxin and CPA. The strains were also reported to produce sclerotia and exhibited dark olive to olive brown colour on CZ agar. A strain was then re-examined for the morphology, mycotoxin production, and the sequences of ITS, β-tubulin and calmodulin gene. Based on these results, a new species named *A. pseudotamarii* was given to replace the previous identification of *A. tamarii* [42]. The characteristics described by [41] are in line with our observation on strain A52R except for the production of CPA. However, the molecular identification based on ITS and β-tubulin were not in agreement with [42], in which the identity of A52R in the present study remains as *A. tamarii* instead of *A. pseudotamarii*. Another study by [14] also reported the presence of *A. tamarii* in peanuts from Argentina, but no aflatoxin was produced by this isolate.

The misidentification of the closely related species in *Aspergillus* section *Flavi* has been reported previously [20]. The authors reported on the misidentification of *A. nomius* and *A. tamarii* as *A. flavus*. According to the authors, this occurred due to the lack of expertise in mycological identification. The similar morphological characteristic of those three species observed on Sabouraud Dextrose Agar (yellow colour) led to the identification of *A. flavus*. However, the sequencing of β-tubulin and calmodulin gene finally and unambiguously identified the species as *A. nomius* and *A. tamarii*. Their finding was also supported by the metabolic fingerprinting, in which *A. flavus*, *A. tamarii* and *A. nomius* were separated into three clusters based on the UHPLC-MS analysis.

*A. arachidicola* and *A. minisclerotigenes*, which were first isolated from the Argentinean peanuts, are known as the closely related species to *A. parasiticus* and *A. flavus*, respectively, and they were also reported to produce AFB, AFG and aspergillic acid [14]. However, none of these species were recorded from peanut samples in the present study even though some strains exhibited similar morphological and chemical characteristics as reported by the author. This indicated that the geographical area might be one of the factors that determine the type of *Aspergillus* spp. that colonise peanuts in fields. In the present study, the raw peanut samples marketed in Malaysia were mostly imported from other countries such as India, China and Vietnam. None of them were from Argentina. 

Based on the phylogenetic analysis, both aflatoxigenic and non-aflatoxigenic *A. flavus* could not be differentiated based on the sequences of ITS and β-tubulin sequences. They were grouped in the same clade except for *A. tamarii* that formed a separate clade. The current results are supported by a previous study on *A. flavus* population from maize [43] and chestnut [22] in Italy, which reported that *A. flavus* was the main species responsible for aflatoxin contamination, and both aflatoxigenic and non-aflatoxigenic strains were also grouped in the same clade. 

Molecular analysis on the aflatoxin biosynthesis gene cluster has proven to be most useful to differentiate the aflatoxigenic and non-aflatoxigenic strains of *A. flavus*. In recent decades, aflatoxin biosynthesis genes have been targeted for the detection of aflatoxigenic fungi in food samples, as the presence of these genes is compulsory for the synthesis of aflatoxin [9,43,44,45]. According to [43], the variability in the aflatoxin gene cluster that exists in the *A. flavus* population is useful in order to understand the risk of aflatoxin contamination as well as the selection of biocontrol agents.

Two sets of multiplex PCRs were used in the present study to detect the presence of aflatoxin biosynthesis genes that code for proteins involved in the aflatoxin biosynthetic pathway at the early stage (*aflD and pksA*), middle stage (*aflM*), and the late stage (*aflP*), and in the regulatory gene (*aflR*) that plays an important role in controlling structural gene expressions [46]. All genes were successfully amplified in the aflatoxigenic *A. flavus* strains (Chemotypes I, II, V), while the non-aflatoxigenic strains failed to amplify at least one of the targeted genes except for a few strains (Chemotype IV). The present findings are in agreement with previous studies [9,44,45,47]. According to [48], the non-aflatoxigenic fungi have varyious amplification patterns. This was further supported by [43] who successfully grouped the non-aflatoxigenic strains into four different amplification patterns. 

In contrast, *A. flavus* strains in Chemotype III were unable to produce aflatoxin even though all the genes were present. Similar findings were also demonstrated by previous researchers [43,48]. The authors suggested that other genes involved in the aflatoxin biosynthesis (which was not tested in the present study) might be lacking or carry some deletions. Chang et al. [29] studied the deletions of a part or the entire aflatoxin biosynthesis gene cluster in non-aflatoxigenic *A. flavus* and suggested that small deletions or mutations in the related genes such as those involved in the signalling pathway or with a regulatory role might have inactivated the aflatoxin biosynthesis pathway of these strains. Besides, the expression of these genes is crucial in determining their ability to produce aflatoxin, as the protein (enzymes) coded by these genes is needed to catalyse the conversion of each aflatoxin precursors. However, gene expression varied among the *A. flavus* strains depending on the physiological and environmental conditions [46,49]. A study by [46] demonstrated a significant difference between *aflD* gene expression at three water activity (a_w_) levels in which a higher expression was observed at 0.90 a_w_ as compared to 0.95 a_w_, and no expression occurred at 0.85 a_w_. *aflD* gene expression was also reported as a reliable marker to differentiate between aflatoxigenic and non-aflatoxigenic *A. flavus* [50]. Besides, the authors suggested to grow the non-aflatoxigenic *A. flavus* strain on the natural food matrix in order to confirm their aflatoxigenic potential.

According to [48], simple mutations (substitution of some bases) could lead to the formation of non-functional products. For example, the *aflR* gene is a regulatory gene and plays an important role in regulating the activity of other structural genes such as *aflP* (*omtA*), *aflD* (*nor-1*) and *aflM* (*ver-1*), and any mutations occurring in the gene will produce a non-functional AFLR gene product that fails to regulate the expression of the structural gene. As a result, no aflatoxin will be produced. The a*flR* gene is also present in some strains of *A. oryzae* and *A. sojae* despite having no record of aflatoxin production [28]. However, the sequences of the amplified *aflR* gene, which was named *A. oryzae*-type *aflR*, showed a consistent variation and can be distinguished from *A. flavus.* It was postulated that this change might affect the DNA-binding capacity of the AFLR protein and disrupt the aflatoxin biosynthesis. 

In the present study, *A. flavus* was the predominant species from section *Flavi* that was found in raw peanut kernel samples collected from all stakeholders along the supply chain. The findings are in line with a previous study by [51], who reported the predominance of *A. flavus* in peanuts from the Busia and Homa bay districts of Western Kenya. Another study by [52] also reported that *A. flavus* was the dominant species found in peanuts during storage. *A. flavus* was able to survive even after the peanuts had been dried prior to storage to reach the moisture content level of less than 11% before they were packed for export. The occurrence of *A. flavus* in the imported peanuts as reported in the present study has proven the survival of its conidia or sclerotia in dried peanut kernels. In contrast, *A. parasiticus* is more dominant in soils from the peanut field as reported by [53], and this might explain the absence of *A. parasiticus* in the present study.

The surveillance and enforcement conducted on the imported raw peanuts by the authorities are only focusing on the aflatoxin level but not the aflatoxigenic fungi that are responsible for aflatoxin production. Thus, the presence of aflatoxin in peanuts at any points along the supply chain, mainly with the manufacturers and retailers, could be due to contamination during storage. The favourable storage conditions are the main cause for the conidia or sclerotia from the aflatoxigenic *A. flavus* to germinate, grow and subsequently produce aflatoxins [54,55]. Moreover, *A. flavus* was also reported in peanut-based products, which demonstrated its ability to invade processed food [8,37]. Thus, the presence of aflatoxin in peanut-based products could be explained by the accumulation and carryover of aflatoxin from raw peanuts or post-contamination of *A. flavus* in the product itself, especially during storage. 

## 4. Conclusions

Molecular analyses on the DNA sequences of ITS and β-tubulin genes have confirmed that *A. flavus* was the only species in section *Flavi* that contaminated raw peanuts and peanut-based products in this study except for one isolate of *A. tamarii.* The phylogenetic analysis grouped all *A. flavus* strains from Chemotypes I–V in the same clade, and *A. tamarii* in a separate clade. In addition, the aflatoxigenic and non-aflatoxigenic *A. flavus* have been described based on the molecular analysis of the aflatoxin biosynthesis genes, *aflR, aflP (omtA), aflD (nor-1), aflM (ver-1)* and *pksA*, in which the results are in line with the aflatoxin production that was described in the previous study [18] except for *A. flavus* strains in Chemotype III. The non-aflatoxigenic *A. flavus* showed varying amplification patterns, which are related to the inability of these isolates to produce aflatoxin.

## 5. Materials and Methods

### 5.1. Fungal Isolates

A total of 77 out of 128 aflatoxigenic and non-aflatoxigenic *Aspergillus* section *Flavi* strains (morphologically identified as *A. flavus* and *A. nomius*) isolated from raw peanuts and peanut-based products from the previous study [18] were used for molecular identification and characterisation in the present study. The source of isolation, chemotype groups, and the GenBank accession number are listed in Table 1. The strains used in this study have been characterised previously using a morphological and chemical approach in which all strains were grouped into five different chemotype profiles depending on the production of B- and G-group aflatoxins, aspergillic acid, and cyclopiazonic acid (CPA). All strains consistently produced aspergillic acid, which was indicated by the orange colour on the reverse of AFPA media. However, the production of aflatoxins and CPA varied and were classified into six different chemotype profiles: Chemotype I (AFB and CPA), Chemotype II (AFB), Chemotype III (CPA), Chemotype IV (none), Chemotype V (AFB, AFG and CPA), and Chemotype VI (AFG). A reference culture of *A. flavus* (NRRL 3357) was used as a positive control. Fungal isolates were sub-cultured on PDA slant and incubated at 30 °C for seven days to enhance the growth and sporulation before they were refrigerated at 4 °C for further use. 

### 5.2. Molecular Identification of Aspergillus Section Flavi

#### 5.2.1. Genomic DNA Extraction

Fungal mycelia for genomic DNA extraction were prepared by inoculating the fungal conidia in in a 150-mL Erlenmeyer flask containing 50 mL Potato Dextrose Broth (PDB) for seven days with shaking at 150 rpm and 30 °C. The mycelia were then filtered using sterile filter paper No. 1 (Whatman, Maidstone, England) and dried under laminar flow. The dried mycelia were subsequently ground to fine powder in liquid nitrogen using a mortar and pestle. The powdered mycelia were weighed and transferred into a 1.5-mL microcentrifuge tube. The genomic DNA extraction was performed by using the DNeasy Plant MiniKit (QIAGEN, Hilden, Germany), following the manufacturer’s instructions, and the purified DNA was kept at −20 °C until further use.

#### 5.2.2. PCR Amplification and Sequencing of ITS and β-Tubulin Genes

The primer pairs used for the ITS region and β-tubulin gene are listed in Table 2. The amplification reaction was carried out in a 25 µL reaction containing 1.0 µL of template DNA (~100 ng), 12.5 µL of EnonoTaq Plus Green 2× Master Mix (Lucigen, Middleton, WI, USA), 2.5 µL of each primer (1.0 µM) (MyTACG Bioscience Enterprise, Selangor, Malaysia) and 6.5 µL of sterile dH_2_O from Elga PureLab Water Purification System (Elga LabWater, High Wycombe, UK). The Master Mix contains the following materials: 0.1 units/µL of EconoTaq DNA Polymerase, Reaction Buffer (pH 9.0), 400 µM dATP, 400 dGTP, 400 dCTP, 400 dATP, 3 mM MgCl_2_, and blue and yellow tracking dyes. The negative control was prepared by using sterile dH_2_O to replace the fungal DNA template. The PCR amplification was performed by using a Veriti Thermal Cycler machine (Applied Biosystems, Waltham, MA, USA). A PCR program for each primer was optimised by using gradient PCR. The optimised condition was as follows: an initial step at 95 °C for one minute, followed by 35 cycles of denaturation at 95 °C for one minute, annealing (at 55 °C for ITS and 61 °C for β-tubulin) for one minute, extension at 72 °C for one minute, and final extension at 72 °C for five minutes. Next, 5 µL of PCR product was loaded in the well and a 100-bp DNA ladder (GeneDireX, Taiwan) was used as a comparison to estimate the size of the PCR product. Gel electrophoresis was conducted by using 1.5% agarose gel (1^st^ Base, Selangor, Malaysia) stained with 0.01% ethidium bromide (Vivantis Technologies, Selangor, Malaysia), and run for 30 min (100 V, 400 mA) using 1× Tris Borate-Ethylenediaminetetraacetic acid, EDTA (TBE) Buffer (1^st^ Base, Selangor, Malaysia). The gel was visualised under UV light and captured using a gel documentation system (SynGene, Cambridge, UK). The PCR products were sent for DNA purification and sequencing to a local service provider (MyTACG Bioscience Enterprise, Selangor, Malaysia).

#### 5.2.3. Sequence Alignment and Species Identification

Following sequencing, consensus sequences were obtained by aligning and editing the forward and reverse sequences using ClustalW in Molecular Evolution and Genetic Analysis (MEGA 7) 2016 software [56]. The consensus sequences were then used to compare with the existing sequences in the GenBank database (http://www.ncbi.nlm.nih.gov) using the Basic Local Alignment Search Tool (BLAST). The identity of isolates was determined by the closest matches between the query and existing sequence from the BLAST search and presented as percentage match of similarity (from 99% to 100%). 

### 5.3. Phylogenetic Analysis

Multiple sequence alignment and phylogenetic analysis was performed using MEGA 7 2016 software [56]. The Maximum Likelihood (ML) method was used on individual and combined ITS and β-tubulin sequences to construct the phylogenetic tree. The ex-type for eight species of *Aspergillus* section *Flavi* as listed in Table 1 were downloaded from the GenBank and included in the phylogenetic analysis for comparison with the current isolates. *A. niger* CBS 113.56 was used as the outgroup. A model test was run to determine the best substitution DNA models with the lowest Akaike Information Criterion (AIC) scores. Tamura 3-parameter model was used to construct the ML tree and the tree reliability was estimated using the bootstrap method with 1000 replicates. Gaps and missing data were treated as complete deletion and excluded from the analysis. A total of 430, 389, and 819 nucleotide characters in the final dataset of individual ITS, β-tubulin, and combined sequences were used in constructing the ML tree respectively.

### 5.4. PCR Amplification and Detection of Aflatoxin Biosynthesis Genes 

Five genes, namely the *aflR, aflP (omtA), aflD (nor-1), aflM (ver-1)* and *pksA* genes, from the aflatoxin biosynthesis cluster and one sugar utilisation gene (*glcA*), as listed previously in Table 3, were amplified using two sets of multiplex PCRs as shown in Table 4. A gradient PCR was used to optimise the annealing temperature from 60–70 °C. 

## Figures and Tables

**Table 1:** GenBank accession numbers of the *Aspergillus* section *Flavi* strains used.

Isolate No.	Species	Stakeholder	* Source	** Chemotype	GenBank Accession Number	
					ITS	β-Tubulin
A8R	*A. flavus*	Manufacturer	Raw peanut kernel (China)	I	MN095114	MN148806
A34R	*A. flavus*	Manufacturer	Raw peanut kernel (India)	I	MN095115	MN148807
A35R	*A. flavus*	Manufacturer	Raw peanut kernel (India)	I	MN095116	MN148808
A42R	*A. flavus*	Manufacturer	Raw peanut kernel (India)	I	MN095117	MN148809
A45R	*A. flavus*	Manufacturer	Raw peanut kernel (India)	I	MN095118	MN148810
A46R	*A. flavus*	Manufacturer	Raw peanut kernel (India)	I	MN095119	MN148811
A47R	*A. flavus*	Manufacturer	Raw peanut kernel (India)	I	MN095120	MN148812
A50R	*A. flavus*	Manufacturer	Raw peanut kernel (China)	I	MN095121	MN148813
A53R	*A. flavus*	Manufacturer	Raw peanut kernel (China)	I	MN095122	MN148814
A54R	*A. flavus*	Manufacturer	Raw peanut kernel (China)	I	MN095123	MN148815
A55R	*A. flavus*	Manufacturer	Raw peanut kernel (China)	I	MN095124	MN148816
A57R	*A. flavus*	Manufacturer	Raw peanut kernel (India)	I	MN095125	MN148817
A58R	*A. flavus*	Manufacturer	Raw peanut kernel (India)	I	MN095126	MN148818
A59R	*A. flavus*	Manufacturer	Raw peanut kernel (India)	I	MN095127	MN148819
A60R	*A. flavus*	Manufacturer	Raw peanut kernel (India)	I	MN095128	MN148820
A61R	*A. flavus*	Manufacturer	Raw peanut kernel (India)	I	MN095129	MN148821
A63R	*A. flavus*	Manufacturer	Raw peanut kernel (India)	I	MN095130	MN148822
A68R	*A. flavus*	Manufacturer	Raw peanut kernel (India)	I	MN095131	MN148823
A74R	*A. flavus*	Manufacturer	Raw peanut kernel (India)	I	MN095132	MN148824
A81R	*A. flavus*	Manufacturer	Raw peanut kernel (China)	I	MN095133	MN148825
A90R	*A. flavus*	Retailer	Raw peanut kernel (India)	I	MN095134	MN148826
A91R	*A. flavus*	Retailer	Raw peanut kernel (India)	I	MN095135	MN148827
A92R	*A. flavus*	Retailer	Raw peanut kernel (India)	I	MN095136	MN148828
A95R	*A. flavus*	Retailer	Raw peanut kernel (India)	I	MN095137	MN148829
A96R	*A. flavus*	Retailer	Raw peanut kernel (India)	I	MN095138	MN148830
A116P	*A. flavus*	Retailer	Peanut-based product (roasted peanut)	I	MN095139	MN148831
A87R	*A. flavus*	Manufacturer	Raw peanut kernel (China)	II	MN095140	MN148832
A88R	*A. flavus*	Manufacturer	Raw peanut kernel (China)	II	MN095141	MN148833
A5R	*A. flavus*	Importer	Raw peanut kernel (China)	III	MN095142	MN148834
A24R	*A. flavus*	Importer	Raw peanut kernel (China)	III	MN095143	MN148835
A40R	*A. flavus*	Manufacturer	Raw peanut kernel (India)	III	MN095144	MN148836
A43R	*A. flavus*	Manufacturer	Raw peanut kernel (China)	III	MN095145	MN148837
A48R	*A. flavus*	Manufacturer	Raw peanut kernel (China)	III	MN095146	MN148838
A49R	*A. flavus*	Manufacturer	Raw peanut kernel (China)	III	MN095147	MN148839
A71R	*A. flavus*	Manufacturer	Raw peanut kernel (India)	III	MN095148	MN148840
A73R	*A. flavus*	Manufacturer	Raw peanut kernel (India)	III	MN095149	MN148841
A77R	*A. flavus*	Importer	Raw peanut in shell (Indonesia)	III	MN095150	MN148842
A78R	*A. flavus*	Manufacturer	Raw peanut kernel (China)	III	MN095151	MN148843
A94R	*A. flavus*	Retailer	Raw peanut kernel (India)	III	MN095152	MN148844
A97R	*A. flavus*	Retailer	Raw peanut kernel (India)	III	MN095153	MN148845
A108P	*A. flavus*	Manufacturer	Peanut-based product (roasted peanut)	III	MN095154	MN148846
A109P	*A. flavus*	Retailer	Peanut-based product (peanut candy)	III	MN095155	MN148847
A118P	*A. flavus*	Retailer	Peanut-based product (roasted peanut)	III	MN095156	MN148848
A9R	*A. flavus*	Retailer	Raw peanut kernel (China)	IV	MN095157	MN148849
A12R	*A. flavus*	Importer	Raw peanut kernel (China)	IV	MN095158	MN148850
A13R	*A. flavus*	Importer	Raw peanut kernel (China)	IV	MN095159	MN148851
A14R	*A. flavus*	Importer	Raw peanut kernel (China)	IV	MN095160	MN148852
A16R	*A. flavus*	Importer	Raw peanut in shell (Vietnam)	IV	MN095161	MN148853
A19R	*A. flavus*	Importer	Raw peanut in shell (China)	IV	MN095162	MN148854
A20R	*A. flavus*	Importer	Raw peanut in shell (China)	IV	MN095163	MN148855
A21R	*A. flavus*	Importer	Raw peanut kernel (China)	IV	MN095164	MN148856
A22R	*A. flavus*	Importer	Raw peanut kernel (China)	IV	MN095165	MN148857
A23R	*A. flavus*	Importer	Raw peanut kernel (China)	IV	MN095166	MN148858
A26R	*A. flavus*	Importer	Raw peanut kernel (China)	IV	MN095167	MN148859
A27R	*A. flavus*	Importer	Raw peanut kernel (China)	IV	MN095168	MN148860
A67R	*A. flavus*	Manufacturer	Raw peanut kernel (India)	IV	MN095169	MN148861
A75R	*A. flavus*	Importer	Raw peanut in shell (Vietnam)	IV	MN095170	MN148862
A76R	*A. flavus*	Importer	Raw peanut in shell (Vietnam)	IV	MN095171	MN148863
A98P	*A. flavus*	Manufacturer	Peanut-based product (peanut snack)	IV	MN095172	MN148864
A104P	*A. flavus*	Manufacturer	Peanut-based product (peanut sauce)	IV	MN095173	MN148865
A111P	*A. flavus*	Retailer	Peanut-based product (peanut snack)	IV	MN095174	MN148866
A114P	*A. flavus*	Retailer	Peanut-based product (roasted peanut)	IV	MN095175	MN148867
A115P	*A. flavus*	Retailer	Peanut-based product (roasted peanut)	IV	MN095176	MN148868
A122R	*A. flavus*	Retailer	Raw peanut kernel (India)	IV	MN095177	MN148869
A123R	*A. flavus*	Retailer	Raw peanut kernel (India)	IV	MN095178	MN148870
A1R	*A. flavus*	Importer	Raw peanut kernel (India)	V	MN095179	MN148871
A15R	*A. flavus*	Importer	Raw peanut kernel (China)	V	MN095180	MN148872
A25R	*A. flavus*	Importer	Raw peanut kernel (China)	V	MN095181	MN148873
A29R	*A. flavus*	Importer	Raw peanut in shell (China)	V	MN095182	MN148874
A41R	*A. flavus*	Manufacturer	Raw peanut kernel (India)	V	MN095183	MN148875
A44R	*A. flavus*	Manufacturer	Raw peanut kernel (India)	V	MN095184	MN148876
A69R	*A. flavus*	Manufacturer	Raw peanut kernel (India)	V	MN095185	MN148877
A80R	*A. flavus*	Manufacturer	Raw peanut kernel (China)	V	MN095186	MN148878
A82R	*A. flavus*	Manufacturer	Raw peanut kernel (China)	V	MN095187	MN148879
A102P	*A. flavus*	Manufacturer	Peanut-based product (peanut cookies)	V	MN095188	MN148880
A107P	*A. flavus*	Manufacturer	Peanut-based product (peanut cookies)	V	MN095189	MN148881
A52R	*A. tamarii*	Manufacturer	Raw peanut in shell (China)	VI	MN095190	MN148882
*A. flavus* NRRL 3357			n.a	n.a	MF966967	M38265
*A. oryzae* CBS 100925			n.a	n.a	KJ175432	EF203138
*A. minisclerotigenes* NRRL 29000			n.a	n.a	KY937929	KY924668
*A. minisclerotigenes* CBS 117620			n.a	n.a	JF422073	EF203150
*A. parvisclerotigenus* CBS 121.62			n.a	n.a	EF409240	EF203130
*A. parasiticus* CBS 100926			n.a	n.a	KJ175437	KJ175497
*A. parasiticus* CBS 100308			n.a	n.a	KJ175436	KJ175496
*A. nomius* NRRL 25393			n.a	n.a	AF027864	AF255068
*A. nomius* NRRL 13137			n.a	n.a	AF027860	AF255067
*A. tamarii* CBS 121599			n.a	n.a	KJ175443	KJ175501
*A. tamarii* CBS 118098			n.a	n.a	KJ175442	KJ175500
*A. arachidicola* CBS 117610			n.a	n.a	EF409241	EF203158
*A. arachidicola* CBS 117614			n.a	n.a	KY937923	KY924665
*A. niger* CBS 113.46			n.a	n.a	FJ629351	FJ629302

* Source: Type of peanuts (County of origin or the type of peanut-based products); n.a: not applicable; ** Chemotype I (AFB, CPA), Chemotype II (AFB), Chemotype III (CPA), Chemotype IV (none), Chemotype V (AFB, AFG, CPA), Chemotype VI (AFB and AFG) [18].

**Table 2:** Amplification pattern of aflatoxin biosynthesis and sugar utilisation genes in *Aspergillus* section *Flavi* strains.

No.	Strain	* Chemotype	Aflatoxin Biosynthesis Gene					
			*aflR*	*aflP (omtA)*	*aflD (nor-1)*	*aflM (ver-1)*	*pksA*	*glcA*
1	A8R	I	+	+	+	+	+	+
2	A34R	I	+	+	+	+	+	+
3	A35R	I	+	+	+	+	+	+
4	A42R	I	+	+	+	+	+	+
5	A45R	I	+	+	+	+	+	+
6	A46R	I	+	+	+	+	+	+
7	A47R	I	+	+	+	+	+	+
8	A50R	I	+	+	+	+	+	+
9	A53R	I	+	+	+	+	+	+
10	A54R	I	+	+	+	+	+	+
11	A55R	I	+	+	+	+	+	+
12	A57R	I	+	+	+	+	+	+
13	A58R	I	+	+	+	+	+	+
14	A59R	I	+	+	+	+	+	+
15	A60R	I	+	+	+	+	+	+
16	A61R	I	+	+	+	+	+	+
17	A63R	I	+	+	+	+	+	+
18	A68R	I	+	+	+	+	+	+
19	A74R	I	+	+	+	+	+	+
20	A81R	I	+	+	+	+	+	+
21	A90R	I	+	+	+	+	+	+
22	A91R	I	+	+	+	+	+	+
23	A92R	I	+	+	+	+	+	+
24	A95R	I	+	+	+	+	+	+
25	A96R	I	+	+	+	+	+	+
26	A116P	I	+	+	+	+	+	+
27	A87R	II	+	+	+	+	+	+
28	A88R	II	+	+	+	+	+	+
29	A5R	III	+	+	+	+	+	+
30	A24R	III	+	+	+	+	+	+
31	A40R	III	+	+	+	−	+	+
32	A43R	III	+	+	+	−	+	+
33	A48R	III	+	+	+	−	+	+
34	A49R	III	+	+	+	+	+	+
35	A71R	III	+	+	+	+	+	+
36	A73R	III	+	+	+	+	+	+
37	A77R	III	+	+	+	+	+	+
38	A78R	III	+	+	+	+	+	+
39	A94R	III	+	+	+	+	+	+
40	A97R	III	+	+	+	+	+	+
41	A108P	III	+	−	+	+	+	+
42	A109P	III	+	+	+	+	+	+
43	A118P	III	+	+	+	+	+	+
44	A9R	IV	−	−	−	−	−	+
45	A12R	IV	−	−	−	−	−	+
46	A13R	IV	−	−	−	−	−	+
47	A14R	IV	−	−	−	−	−	+
48	A16R	IV	−	−	−	−	−	+
49	A19R	IV	−	−	−	−	−	+
50	A20R	IV	−	−	−	−	−	+
51	A21R	IV	−	−	−	−	−	+
52	A22R	IV	−	−	−	−	−	+
53	A23R	IV	+	+	+	+	+	+
54	A26R	IV	−	−	−	−	−	+
55	A27R	IV	−	−	−	−	−	+
56	A67R	IV	+	+	+	+	+	+
57	A75R	IV	−	−	−	−	−	+
58	A76R	IV	−	−	−	−	−	+
59	A98P	IV	−	−	−	−	−	+
60	A104P	IV	−	+	−	+	−	+
61	A111P	IV	−	−	−	−	−	+
62	A114P	IV	−	+	+	+	+	+
63	A115P	IV	−	−	−	−	−	+
64	A122R	IV	+	+	+	+	+	+
65	A123R	IV	+	+	+	+	+	+
66	A1R	V	+	+	+	+	+	+
67	A15R	V	+	+	+	+	+	+
68	A25R	V	+	+	+	+	+	+
69	A29R	V	+	+	+	+	+	+
70	A41R	V	+	+	+	+	+	+
71	A44R	V	+	+	+	+	+	+
72	A69R	V	+	+	+	+	+	+
73	A80R	V	+	+	+	+	+	+
74	A82R	V	+	+	+	+	+	+
75	A102P	V	+	+	+	+	+	+
76	A107P	V	+	+	+	+	+	+
77	A52R	VI	+	+	+	+	+	+

+ present; − absent; * Chemotype I (AFB, CPA), Chemotype II (AFB), Chemotype III (CPA), Chemotype IV (none), Chemotype V (AFB, AFG, CPA), Chemotype VI (AFG) [18].

**Table 3:** List of primers used for DNA sequencing, aflatoxin biosynthesis genes and sugar utilisation gene detection.

Target Gene	Primer	Primer Sequences	Size (bp)	References
ITS region	ITS 1	5′-TCC GTA GGT GAA CCT GCG G-3′	600	[57,58]
	ITS 4	5′-TCC TCC GCT TAT TGA TAT GC-3′		
β-tubulin	Bt2a	5′- GGT AAC CAA ATC GGT GCT GCT TTC-3′	495	[57]
	Bt2b	5′-ACC CTC AGT GTA GTG ACC CTT GGC-3′		
*aflR*	*aflr1*	5′-TAT CTC CCC CCG GGC ATC TCC CGG-3′	1032	[48,59]
	*aflr2*	5′-CCG TCA GAC AGC CAC TGG ACA CGG-3′		
*aflP (omtA)*	*omt1*	5′-GGC CCG GTT CCT TGG CTC CTA AGC-3′	1024	[59]
	*omt2*	5′-CGC CCC AGT GAG ACC CTT CCT CG-3′		
*aflD (nor-1)*	*nor1*	5′-ACC GCT ACG CCG GCA CTC TCG GCA C-3′	400	[48]
	*nor2*	5′-GTT GGC CGC CAG CTT CGA CAC TCC G-3′		
*aflM (ver-1)*	*ver1*	5′-GCC GCA GGC CGC GGA GAA AGT GGT-3′	537	[48]
	*ver2*	5′-GGG GAT ATA CTC CCG CGA CAC AGC C-3′		
*pksA*	*pksa1*	5′-GCT GGG ATT CTG CAT GGG TT-3′	536	[30]
	*pksa2*	5′-CAG TTG CTC CCA AGG AGT GGT-3′		
*glcA*	*glca1*	5′-GTA CGA TGC AAA TGG CGT CC-3′	851	[60]
	*glca2*	5′-GAA GCT CTG TGT CGT TGG GA-3′		

**Table 4:** Multiplex PCR condition.

Set	Primers	PCR Reaction Condition	Cycle
1	*omt1/omt2*, *pksa1/pksa2*, *glca1/glca2*	Initial denaturation: 95 °C, 1 min Denaturation: 95 °C, 1 min Annealing: 61 °C, 1 min Extension: 72 °C, 1 min Final extension: 72 °C, 5 min	1 30 30 30 1
2	*aflr1/aflr2*, *ver1/ver2*, *nor1/nor2*	Initial denaturation: 95 °C, 1 min Denaturation: 95 °C, 1 min Annealing: 67 °C, 1 min Extension: 72 °C, 1 min Final extension: 72 °C, 5 min	1 30 30 30 1
